# Supplementary material for: Context-Based Facilitation in Visual Word Recognition: Evidence for Visual and Lexical But Not Pre-Lexical Contributions
Source: eNeuro. 2019 May 8;6(2):ENEURO.0321-18.2019. doi: 10.1523/ENEURO.0321-18.2019 (PMC6509571; doi:10.1523/ENEURO.0321-18.2019)
Supplement: Extended Data Figure 3-1 — Results from the LMM analyses of d’ sensitivity indices from the PW familiarization sessions of both experiments 1 and 2, including pairwise comparisons from session to session. Download Figure 3-1, DOCX file. [file sup_enu-eN-NWR-0321-18-s07.docx]

| *Figure 3-1.* Results from the linear mixed model analyses of d' sensitivity indices from the pseudoword familiarization sessions of both Experiment 1 and 2, including pairwise comparisons from session to session. | | | | | | | | | | | |
| --- | --- | --- | --- | --- | --- | --- | --- | --- | --- | --- | --- |
|  | Experiment 1 | | |  | Experiment 2 | | | | | | |
|  | Old/New recognition | | |  | Paired-association | | |  | Naming | | |
|  | *FE* | *SE* | *t* |  | *FE* | *SE* | *t* |  | *FE* | *SE* | *t* |
| All Sessions | **0.66** | **0.039** | **17.17** |  | **0.57** | **0.033** | **17.11** |  | **0.77** | **0.040** | **19.41** |
| 1 vs. 2 | **0.50** | **0.060** | **8.26** |  | **0.38** | **0.046** | **8.25** |  | **0.39** | **0.051** | **7.63** |
| 2 vs. 3 | **0.25** | **0.029** | **8.52** |  | **0.22** | **0.026** | **8.61** |  | **0.31** | **0.048** | **6.56** |
| 3 vs. 4 | **0.16** | **0.029** | **5.75** |  | **0.11** | **0.046** | **2.48** |  | **0.21** | **0.041** | **5.13** |
| 4 vs. 5 | - | - | - |  | **0.12** | **0.029** | **4.20** |  | **0.20** | **0.043** | **4.63** |
| *Note*. Significant effects (i.e., *t* > 2) are shown in bold numerals. *FE* = fixed effect estimates. | | | | | | | | | | | |
