# Supplementary material for: Context-Based Facilitation in Visual Word Recognition: Evidence for Visual and Lexical But Not Pre-Lexical Contributions
Source: eNeuro. 2019 May 8;6(2):ENEURO.0321-18.2019. doi: 10.1523/ENEURO.0321-18.2019 (PMC6509571; doi:10.1523/ENEURO.0321-18.2019)
Supplement: Extended Data Figure 4-1 — Overview of clusters demonstrating a prime/target by repetition congruency (repetition vs non-repetition) interaction obtained with separate or common baselines for prime and target. Download Figure 4-1, DOCX file. [file sup_enu-eN-NWR-0321-18-s08.docx]

| *Figure 4-1.* Overview of clusters demonstrating a prime/target by repetition congruency (repetition vs. non-repetition) interaction obtained with separate or common baselines for prime and target. | | | | |
| --- | --- | --- | --- | --- |
| **Cluster** | **Analysis** | **Sensors** | **Time Range** | ***p*** |
| 1 | Separate baselines | **left frontal** | **0.28** to **0.45** | **0.0002** |
|  | Common baseline | left frontal | 0.28 to 0.46 | 0.0002 |
| 2 | Separate baselines | **right frontal** | **0.32** to **0.55** | **0.0002** |
|  | Common baseline | right frontal | 0.29 to 0.5 | 0.0002 |
| 3 | Common baseline | left central | 0.74 to 0.77 | 0.018 |
| 4 | Common baseline | left occipital | 0.36 to 0.38 | 0.039 |
| *Note.* Clusters found in both separate and common baseline analysis presented in the results section are marked in bold. *p* refers to the multiple-comparison-corrected ratio of permuted cluster statistics larger than the cluster statistic of the original data. | | | | |
