# Supplementary figures and images for: Context-Based Facilitation in Visual Word Recognition: Evidence for Visual and Lexical But Not Pre-Lexical Contributions
Source: eNeuro. 2019 May 8;6(2):ENEURO.0321-18.2019. doi: 10.1523/ENEURO.0321-18.2019 (PMC6509571; doi:10.1523/ENEURO.0321-18.2019)

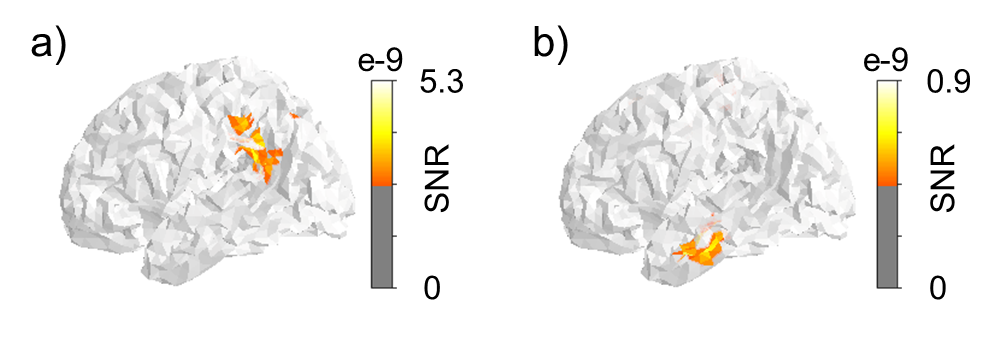

Supplement: Extended Data Figure 5-2 — Source locations for (A) the familiarity cluster F1 contrasting familiar PWs > words and (B) the interaction cluster CxF contrasting prime versus target by words versus familiar PWs. Download Figure 5-2, TIF file. [file sup_enu-eN-NWR-0321-18-s01.tif]

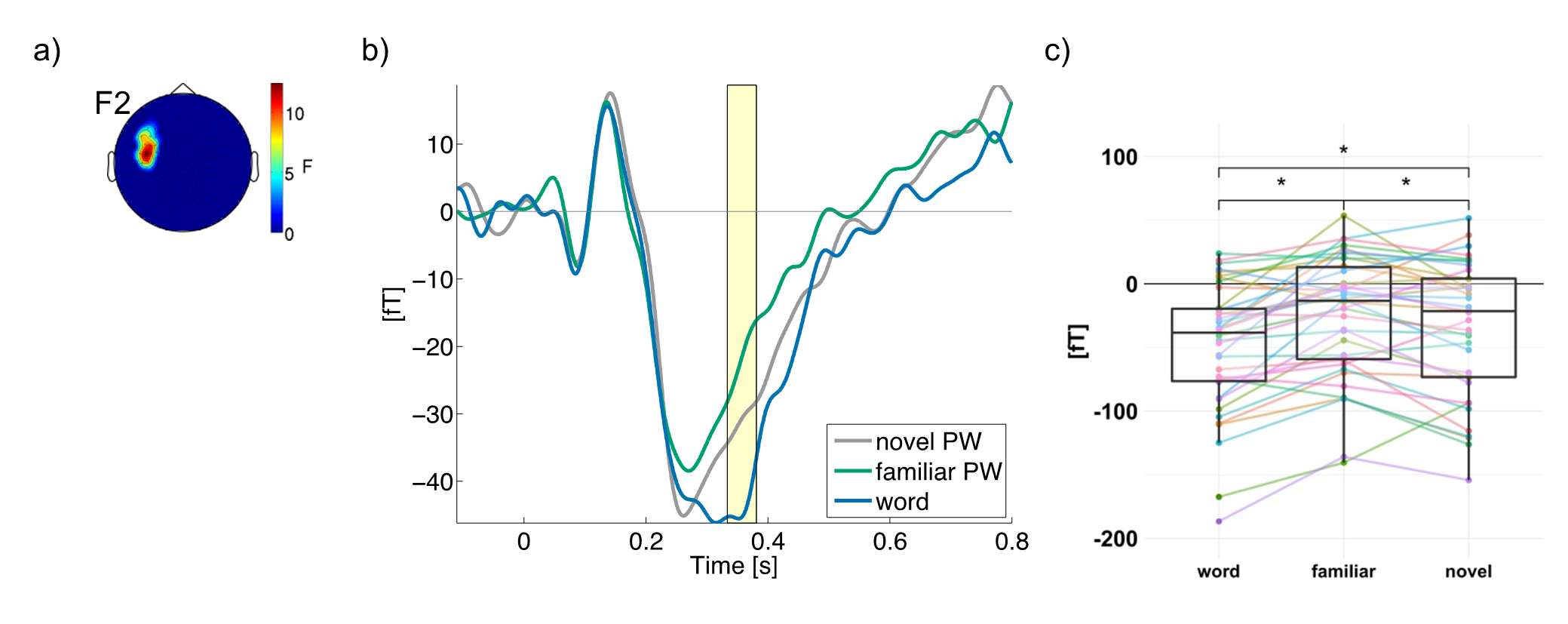

Supplement: Extended Data Figure 5-3 — Main effect of lexical familiarity. Topographical map (A), ERF time course (B), and boxplot (C), averaged across prime and target. Download Figure 5-3, TIF file. [file sup_enu-eN-NWR-0321-18-s02.tif]

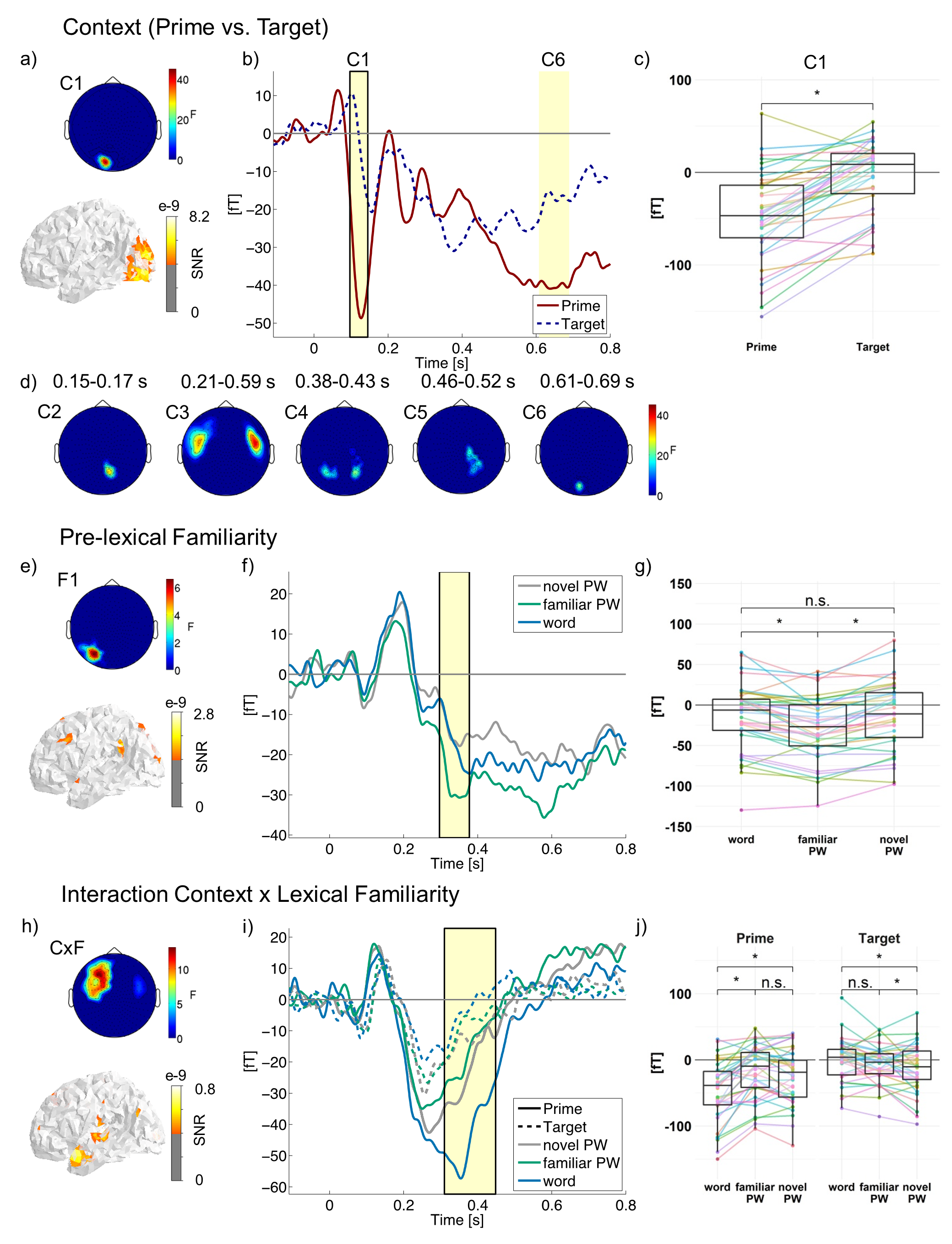

Supplement: Extended Data Figure 5-4 — Same results as in Figure 5 depicted for a low-pass filter of 40 instead of 20 Hz. Download Figure 5-4, TIF file. [file sup_enu-eN-NWR-0321-18-s03.tif]

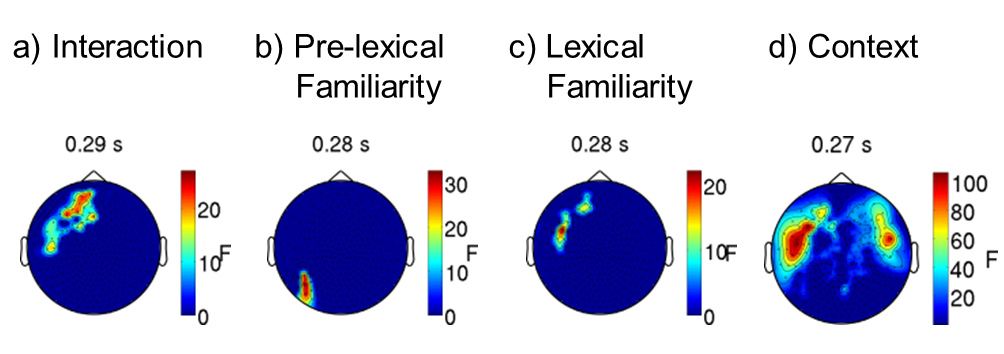

Supplement: Extended Data Figure 5-5 — Significant clusters obtained in the peak-to-peak analysis for (A) the interaction of context (prime vs target) by lexical familiarity, (B) main effects of pre-lexical familiarity, (C) main effects of lexical familiarity, and (D) main effect of context (prime vs target). Topographical maps represent F values of significant sensors; note the different scales. Non-significant sensors are set to zero. Peak latencies averaged across significant sensors and all conditions are depicted above the topographical maps. Download Figure 5-5, TIF file. [file sup_enu-eN-NWR-0321-18-s04.tif]

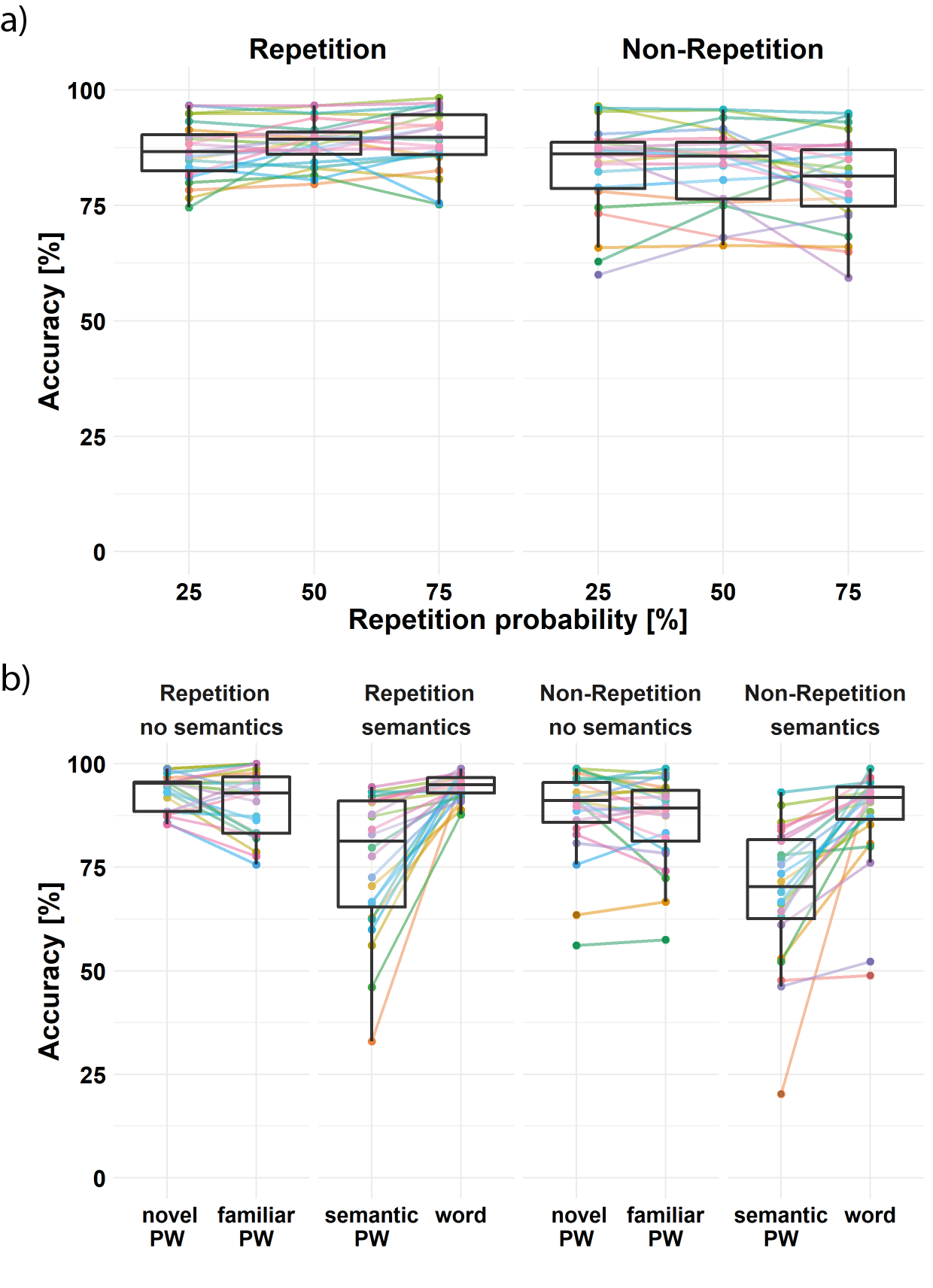

Supplement: Extended Data Figure 6-1 — Accuracies in experiment 2 (semantic association judgment task). A, Repetition probability effect for repetition (left) and non-repetition trials (right) averaged across familiarity conditions. B, Familiarity influence on context effects, i.e., repeated (left) versus non-repeated targets (right). Effects are separated for familiarity conditions, with an additional separation for letter strings with and without semantic associations, averaged across repetition probabilities. Colored dots and lines represent individual participants. Download Figure 6-1, TIF file. [file sup_enu-eN-NWR-0321-18-s05.tif]
