# Supplementary material for: Context-Based Facilitation in Visual Word Recognition: Evidence for Visual and Lexical But Not Pre-Lexical Contributions
Source: eNeuro. 2019 May 8;6(2):ENEURO.0321-18.2019. doi: 10.1523/ENEURO.0321-18.2019 (PMC6509571; doi:10.1523/ENEURO.0321-18.2019)
Supplement: Extended Data Figure 5-1 — Overview of clusters form the analysis investigating the prime/target × familiarity interaction obtained with separate or common baselines for prime and target, as well as peak-to-peak analysis. Download Figure 5-1, DOCX file. [file sup_enu-eN-NWR-0321-18-s09.docx]

| *Figure 5-1.* Overview of clusters form the analysis investigating the prime/target x familiarity interaction obtained with separate or common baselines for prime and target, as well as peak-to-peak analysis. | | | | |
| --- | --- | --- | --- | --- |
| **Cluster** | **Analysis** | **Sensors** | **Time Range** | ***p*** |
| *Prime/Target x Familiarity Interaction* | | | | |
| 1 | Separate baselines | **left frontal** | **0.3 to 0.48** | **0.0002** |
|  | Common baseline | left frontal | 0.29 to 0.48 | 0.0002 |
|  | Peak-to-peak | left frontal | 0.29 | 0.0002 |
| 2 | Separate baselines | **right frontal** | **0.4 to 0.44** | **0.0008** |
|  | Common baseline | right frontal | 0.4 to 0.46 | 0.0004 |
| 3 | Separate baselines | left occipital | 0.3 to 0.33 | 0.0068 |
|  | Peak-to-peak | left occipital | 0.28 | 0.0002 |
| 4* | Separate baselines | left central | 0.27 to 0.29 | 0.033 |
|  | Common baseline | left central | 0.26 to 0.29 | 0.0032 |
| 5 | Common baseline | left central | 0.48 to 0.5 | 0.0054 |
| 6 | Common baseline | right frontal | 0.35-0.38 | 0.014 |
| 7 | Common baseline | right central | 0.32-0.34 | 0.021 |
| 8 | Common baseline | left frontal | 0.01-0.02 | 0.031 |
| 9 | Common baseline | left central | 0.04-0.05 | 0.042 |
| 10 | Common baseline | right central | 0.43-0.45 | 0.042 |
| *Main effect of familiarity* | | | | |
| 1 | Separate baselines | **left occipital** | **0.29 to 0.38** | **0.0002** |
|  | Common baseline | left occipital | 0.3 to 0.34 | **0.0002** |
|  | Peak-to-peak | left occipital | 0.28 | 0.0002 |
| 2 | Separate baselines | **left frontal** | **0.33 to 0.38** | **0.0002** |
|  | Common baseline | left frontal | 0.35 to 0.37 | **0.002** |
|  | Peak-to-peak | left frontal | 0.28 | 0.0002 |
| 3 | Separate baselines | left occipital | 0.43 to 0.48 | 0.0002 |
| 4 | Separate baselines | left occipital | 0.56 to 0.6 | 0.008 |
| 5 | Separate baselines | right central | 0.55 to 0.57 | 0.014 |
| 6 | Common baseline | posterior central | 0.05 | 0.014 |
| 7 | Common baseline | left temporal | -0.11 to -0.1 | 0.008 |
| *Main effect of prime vs. target* | | | | |
| 1 | Separate baselines | **left frontal** | **0.21 to 0.47** | **0.0002** |
|  | Common baseline | left frontal | 0.15 to 0.51 | 0.0002 |
|  | Peak-to-peak | bilateral frontal | 0.27 | 0.0002 |
| 2 | Separate baselines | **right frontal** | **0.26 to 0.59** | **0.0002** |
|  | Common baseline | right frontal | 0.22 to 0.61 | 0.0002 |
|  | Peak-to-peak | *see above* | *see above* | *see above* |
| 3 | Separate baselines | **left occipital** | **0.1 to 0.14** | **0.001** |
|  | Common baseline | left occipital | 0.1 to 0.13 | 0.0076 |
| 4 | Separate baselines | **right central / temporal** | **0.47 to 0.53** | **0.0002** |
|  | Common baseline | right central | 0.48 to 0.52 | 0.011 |
| 5 | Separate baselines | **right central** | **0.38 to 0.42** | **0.0004** |
|  | Common baseline | right central | 0.37 to 0.42 | 0.0032 |
| 6 | Separate baselines | **left temporal** | **0.38 to 0.42** | **0.0034** |
|  | Common baseline | left temporal | 0.37 to 0.44 | 0.0008 |
| 7 | Separate baselines | **right central** | **0.15 to 0.18** | **0.0038** |
|  | Common baseline | right central | 0.15 to 0.18 | 0.0006 |
| 8 | Separate baselines | **left occipital** | **0.59 to 0.69** | **0.0076** |
|  | Common baseline | left occipital | 0.61 to 0.7 | 0.0026 |
| 9* | Separate baselines | right central/ frontal | 0.08 to 0.17 | 0.0002 |
|  | Common baseline | right central | 0.09 to 0.11 | 0.010 |
| 10* | Separate baselines | right occipital | 0.2 to 0.25 | 0.0072 |
|  | Common baseline | right occipital | 0.22 to 0.24 | 0.034 |
| 11 | Separate baselines | left central/ temporal | 0.08 to 0.13 | 0.0002 |
| 12 | Separate baselines | right occipital | 0.1 to 0.13 | 0.017 |
| 13 | Separate baselines | right central | 0.59 to 0.62 | 0.027 |
| 14 | Common baseline | right central | -0.11 to -0.04 | 0.001 |
| 15 | Common baseline | left central | 0.05 to 0.08 | 0.002 |
| 16 | Common baseline | left central | -0.06 to -0.01 | 0.0038 |
| 17 | Common baseline | right central | -0.04 to -0.02 | 0.030 |
| 18 | Common baseline | right frontal | 0.62 to 0.66 | 0.047 |
| *Note.* Clusters found in both separate and common baseline analysis presented in the results section are marked in bold. Asterisks mark clusters that were significant in both analyses but not interpreted due to the short duration < 30 ms. For peak-to-peak analysis, the time range row represents the peak latency averaged across all conditions and significant sensors. *p* refers to the multiple-comparison-corrected ratio of permuted cluster statistics larger than the cluster statistic of the original data. | | | | |
