# Supplementary material for: Context-Based Facilitation in Visual Word Recognition: Evidence for Visual and Lexical But Not Pre-Lexical Contributions
Source: eNeuro. 2019 May 8;6(2):ENEURO.0321-18.2019. doi: 10.1523/ENEURO.0321-18.2019 (PMC6509571; doi:10.1523/ENEURO.0321-18.2019)
Supplement: Extended Data Table 4-1 — Results from post hoc LMM analyses on ERF values (in 10−14 Tesla) from sensor and time point of the strongest effect, separately for prime and target, for pre-lexical (F1) and lexical (F2) familiarity clusters represented in Figure 5D–I and Extended Data Figure 5-4, respectively. Download Table 4-1, DOCX file. [file sup_enu-eN-NWR-0321-18-s10.docx]

| *Table 4-1.* Results from *post hoc* LMM analyses on ERF values (in 10^-14^ Tesla) from sensor and time point of the strongest effect, separately for prime and target, for pre-lexical (F1) and lexical (F2) familiarity clusters represented in Figure 5d-i and Extended Data Figure 5-4, respectively | | | | | | | |
| --- | --- | --- | --- | --- | --- | --- | --- |
|  | Prime | | |  | Target | | |
|  | *FE* | *SE* | *t* |  | *FE* | *SE* | *t* |
|  | *F1* | | | | | | |
|  | Word vs. novel PW | | | | | | |
| Familiarity | **-1.01** | **0.49** | **2.08** |  | **1.30** | **0.45** | **2.90** |
| OLD20 | -0.27 | 0.24 | 1.11 |  | -0.018 | 0.22 | 0.082 |
| Number of syllables | 0.22 | 0.24 | 0.94 |  | 0.16 | 0.22 | 0.75 |
|  | Words vs. familiar PW | | | | | | |
| Familiarity | **-2.63** | **0.47** | **5.60** |  | 0.024 | 0.46 | 0.053 |
| OLD20 | -0.084 | 0.24 | 0.36 |  | 0.013 | 0.23 | 0.056 |
| Number of syllables | 0.037 | 0.22 | 0.17 |  | 0.12 | 0.22 | 0.57 |
|  | Familiar vs. novel PW | | | | | | |
| Familiarity | **1.70** | **0.43** | **4.00** |  | **1.31** | **0.47** | **2.78** |
| OLD20 | -0.047 | 0.21 | -0.22 |  | -0.11 | 0.24 | 0.48 |
| Number of syllables | -0.098 | 0.21 | 0.46 |  | -0.020 | 0.23 | 0.086 |
|  | *F2* | | | | | | |
|  | Word vs. novel PW | | | | | | |
| Familiarity | **3.46** | **0.83** | **4.19** |  | -1.07 | 0.70 | 1.53 |
| OLD20 | -0.12 | 0.41 | 0.30 |  | -0.075 | 0.35 | 0.22 |
| Number of syllables | 0.18 | 0.40 | 0.45 |  | 0.42 | 0.34 | 1.23 |
|  | Words vs. familiar PW | | | | | | |
| Familiarity | **5.38** | **0.80** | **6.74** |  | -0.34 | 0.64 | 0.53 |
| OLD20 | 0.28 | 0.40 | 0.69 |  | -0.32 | 0.32 | 1.00 |
| Number of syllables | 0.39 | 0.37 | 1.06 |  | 0.40 | 0.30 | 1.31 |
|  | Familiar vs. novel PW | | | | | | |
| Familiarity | **-1.66** | **0.67** | **2.47** |  | -0.84 | 0.60 | 1.40 |
| OLD20 | 0.029 | 0.34 | 0.087 |  | -0.36 | 0.30 | 1.22 |
| Number of syllables | -0.087 | 0.33 | 0.26 |  | 0.11 | 0.30 | 0.37 |
| Significant effects (i.e., *t* > 2) are shown in bold numerals. *FE* = fixed effect estimates. | | | | | | | |
