# Supplementary material for: Context-Based Facilitation in Visual Word Recognition: Evidence for Visual and Lexical But Not Pre-Lexical Contributions
Source: eNeuro. 2019 May 8;6(2):ENEURO.0321-18.2019. doi: 10.1523/ENEURO.0321-18.2019 (PMC6509571; doi:10.1523/ENEURO.0321-18.2019)
Supplement: Extended Data Table 5-1 — Results from post hoc LMM analyses on ERF values (in 10−14 Tesla) from sensor and time point of the strongest effect, separately for prime and target, for the prime/target × lexical familiarity interaction cluster represented in Figure 5H–J. Download Table 5-1, DOCX file. [file sup_enu-eN-NWR-0321-18-s11.docx]

| *Table 5-1.* Results from *post hoc* LMM analyses on ERF values (in 10^-14^ Tesla) from sensor and time point of the strongest effect, separately for prime and target, for the prime/target x lexical familiarity interaction cluster represented in Figure 5h-j | | | | | | | |
| --- | --- | --- | --- | --- | --- | --- | --- |
|  | Prime | | |  | Target | | |
|  | *FE* | *SE* | *t* |  | *FE* | *SE* | *t* |
|  | Word vs. novel PW | | | | | | |
| Familiarity | **2.18** | **0.47** | **4.67** |  | **-1.20** | **0.37** | **3.28** |
| OLD20 | -0.11 | 0.23 | 0.46 |  | 0.080 | 0.18 | 0.44 |
| Number of syllables | 0.060 | 0.23 | 0.27 |  | 0.14 | 0.18 | 0.81 |
|  | Words vs. familiar PW | | | | | | |
| Familiarity | **3.11** | **0.41** | **7.56** |  | -0.66 | 0.37 | 1.81 |
| OLD20 | 0.22 | 0.21 | 1.05 |  | -0.16 | 0.18 | 0.90 |
| Number of syllables | 0.11 | 0.19 | 0.59 |  | 0.19 | 0.17 | 1.13 |
|  | Familiar vs. novel PW | | | | | | |
| Familiarity | -0.71 | 0.40 | 1.76 |  | **-0.69** | **0.33** | **2.06** |
| OLD20 | -0.028 | 0.20 | 0.14 |  | -0.059 | 0.17 | 0.35 |
| Number of syllables | -0.071 | 0.20 | 0.36 |  | 0.0036 | 0.17 | 0.022 |
| Significant effects (i.e., *t* > 2) are shown in bold numerals. *FE* = fixed effect estimates. | | | | | | | |
