# Supplementary material for: Context-Based Facilitation in Visual Word Recognition: Evidence for Visual and Lexical But Not Pre-Lexical Contributions
Source: eNeuro. 2019 May 8;6(2):ENEURO.0321-18.2019. doi: 10.1523/ENEURO.0321-18.2019 (PMC6509571; doi:10.1523/ENEURO.0321-18.2019)
Supplement: Extended Data Table 10-1 — Results from the LMM analyses investigating repetition congruency (repetition vs non-repetition), probability, familiarity, and semantics in accuracies during repetition priming (experiment 2). Download Table 10-1, DOCX file. [file sup_enu-eN-NWR-0321-18-s12.docx]

| *Table 10-1.* Results from the linear mixed model analyses investigating repetition congruency (repetition vs. non-repetition), probability, familiarity, and semantics in accuracies during repetition priming (Experiment 2). | | | | |
| --- | --- | --- | --- | --- |
|  | *FE* | *SE* | *z* | *p* |
| Repetition congruency | **0.29** | **0.027** | **10.78** | **< 2e-16** |
| Probability | 0.023 | 0.028 | 0.82 | 0.41 |
| Pre-lexical familiarity | **-0.44** | **0.027** | **16.08** | **< 2e-16** |
| Lexical familiarity | **-0.27** | **0.027** | **-9.8** | **< 2e-16** |
| Repetition congruency x Probability | **0.10** | **0.028** | **3.58** | **0.00034** |
| Repetition congruency x Pre-lexical familiarity | **-0.09** | **0.027** | **3.31** | **0.00093** |
| Repetition congruency x Lexical familiarity | 0.027 | 0.027 | 1.00 | 0.32 |
| Probability x Pre-lexical familiarity | -0.021 | 0.028 | -0.76 | 0.44 |
| Probability x Lexical familiarity | **0.064** | **0.028** | **2.32** | **0.020** |
| Pre-lexical x Lexical familiarity | **-0.28** | **0.027** | **10.38** | **< 2e-16** |
| Repetition congruency x Probability x Pre-lexical familiarity | -0.037 | 0.028 | 1.35 | 0.18 |
| Repetition congruency x Probability x Lexical familiarity | **0.075** | **0.028** | **2.72** | **0.0065** |
| Repetition congruency x Pre-lexical x Lexical familiarity | -0.039 | 0.027 | 1.44 | 0.15 |
| Probability x Pre-lexical x Lexical familiarity | -0.015 | 0.028 | 0.54 | 0.59 |
| OLD20 | **0.071** | **0.023** | **3.06** | **0.0022** |
| Number of syllables | 0.045 | 0.024 | 1.84 | 0.065 |
| *Note*. Significant effects (i.e., *p* < 0.05) are shown in bold numerals. *FE* = fixed effect estimates. | | | | |
