# Supplementary material for: Context-Based Facilitation in Visual Word Recognition: Evidence for Visual and Lexical But Not Pre-Lexical Contributions
Source: eNeuro. 2019 May 8;6(2):ENEURO.0321-18.2019. doi: 10.1523/ENEURO.0321-18.2019 (PMC6509571; doi:10.1523/ENEURO.0321-18.2019)
Supplement: Extended Data Table 10-2 — Results from the LMM analyses on accuracies during repetition priming (experiment 2), separately for repetition and non-repetition trials. Download Table 10-2, DOCX file. [file sup_enu-eN-NWR-0321-18-s13.docx]

| *Table 10-2.* Results from the linear mixed model analyses on accuracies during repetition priming (Experiment 2), separately for repetition and non-repetition trials. | | | | | | | | | |
| --- | --- | --- | --- | --- | --- | --- | --- | --- | --- |
|  | Repetition | | | |  | Non-Repetition | | | |
|  | *FE* | *SE* | *z* | *p* |  | *FE* | *SE* | *z* | *p* |
| Probability | **0.11** | **0.040** | **2.81** | **0.0049** |  | -0.067 | 0.036 | 1.87 | 0.061 |
| Pre-lexical familiarity | **-0.56** | **0.042** | **13.54** | **< 2e-16** |  | **-0.40** | **0.040** | **9.84** | **< 2e-16** |
| Lexical familiarity | **-0.18** | **0.041** | **-4.38** | **1.21e-05** |  | **-0.33** | **0.041** | **-8.02** | **1.02e-15** |
| Probability x Pre-lexical familiarity | -0.053 | 0.039 | 1.34 | 0.18 |  | 0.0037 | 0.034 | 0.11 | 0.91 |
| Probability x Lexical familiarity | **0.13** | **0.040** | **3.24** | **0.0012** |  | -0.0052 | 0.034 | -0.16 | 0.88 |
| Pre-lexical x Lexical familiarity | **-0.36** | **0.042** | **8.66** | **< 2e-16** |  | **-0.30** | **0.040** | **7.53** | **5.19e-14** |
| Probability x Pre-lexical x Lexical familiarity | -0.063 | 0.039 | 1.60 | 0.11 |  | 0.027 | 0.033 | 0.80 | 0.42 |
| OLD20 | **0.10** | **0.036** | **2.77** | **0.0055** |  | 0.051 | 0.050 | 1.03 | 0.31 |
| Number of syllables | 0.073 | 0.040 | 1.82 | 0.069 |  | 0.017 | 0.053 | 0.33 | 0.75 |
| *Note.* Significant effects (i.e., *p* < 0.05) are shown in bold numerals. *FE* = fixed effect estimates. | | | | | | | | | |
